# Spectroscopic investigation of the effects of simulated open waste burning on the functional and surface chemistry of commercial polystyrene

**DOI:** 10.1039/d5va00291e

**Published:** 2026-03-09

**Authors:** Maycee Hurd, Xuewen Wang, Angelica Benavidez, Allyson L. McGaughey, Michael Spilde, José M. Cerrato, Jorge Gonzalez-Estrella, Eliane El Hayek

**Affiliations:** a Gerald May Department of Civil, Construction & Environmental Engineering, University of New Mexico MSC01 1070 Albuquerque New Mexico 87131 USA mhurd@andrew.cmu.edu; b School of Civil & Environmental Engineering, Oklahoma State University 248 Engineering North Stillwater Oklahoma 74078 USA; c Center for Micro Engineered Materials 1001 University Dr Albuquerque New Mexico 87106 USA; d Department of Earth and Planetary Sciences, University of New Mexico MSC03 2040 Albuquerque NM 87131 USA; e Department of Pharmaceutical Sciences, University of New Mexico, College of Pharmacy MSC09 5360 Albuquerque New Mexico 87131 USA

## Abstract

We studied the spectroscopic changes of polystyrene (PS) plastic used for commercial food packaging after thermal oxidation. Thermal oxidation of plastic in the environment occurs during open waste burning (OWB), urban wildfires, and even the serving of hot food. Environmental thermally oxidized plastics are understudied and display unique functional and surface chemistry compared to plastics exposed to other environmental weathering processes. Near-surface X-ray photoelectron spectroscopy (XPS) C 1s high resolution analyses show an increase in the intensity of peaks greater than 285 eV, corresponding to C/O bonds after burning of PS at 350 and 425 °C. Bulk analyses using Attenuated Total Reflectance Fourier Transform Infrared (ATR-FTIR) Spectroscopy show that thermal oxidation decreased the alkene (3040–3000 cm^−1^) and alkane (3000–2850 cm^−1^) C–H stretch signals, suggesting PS chain scission. Morphology defects and fractures were observed in scanning electron microscopy (SEM) images, indicating that the degradation of PS can lead to fragmentation after burning. The outcome of this study furthers the understanding of the thermal oxidative degradation mechanisms during incineration of commercial PS materials, which can inform future risk assessments of public and environmental health associated with OWB.

## Abstract 2

Simulated open waste burning of polystyrene plastics results in significant changes to both bulk and surface chemistry.

## Graphical abstract

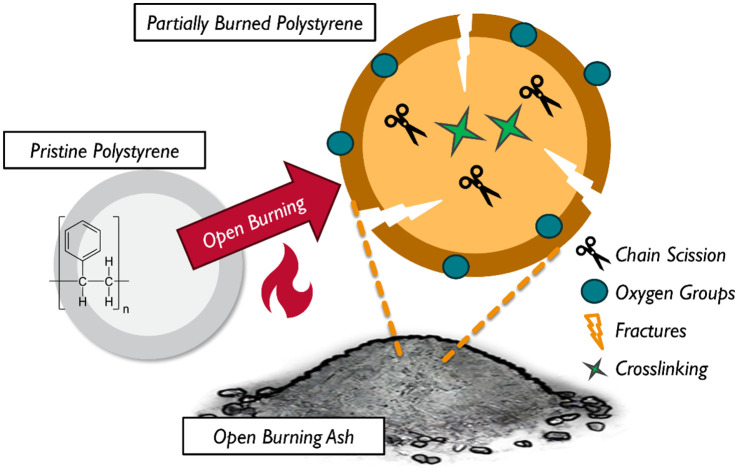

**Environmental significance**

Plastics exposed to high temperatures due to environmental or anthropogenic processes (*e.g.*, wildfires, open burning) are quickly weathered, changing their composition. We identified physical and chemical changes of commercial polystyrene food packaging waste after simulated open burning. These findings enhance our mechanistic understanding of environmentally relevant thermal weathering to inform future soil and water risk assessments. The spectroscopic signatures in this work also aid monitoring efforts of unknown environmental microplastics.

## Introduction

High temperature thermally oxidized plastics are present in the environment and possess unique functional differences from their parent materials. This thermal oxidation can happen both during and after a plastic product's useful life. When in use, commercial food containers experience temperatures and wear great enough to induce degradation^1^ and plastics found in building materials degrade during urban wildfires,^2^ which have significantly increased in frequency over recent years.^3^ At the end-of-life stage, waste incineration and open waste burning (OWB), a common practice used in many developing and underserved communities,^4–6^ directly induces thermal oxidation and leaves the degraded plastics in the ash^7–9^ or open environment.^10,11^ The toxic atmospheric contaminants produced from open burning, including polycyclic aromatic hydrocarbons (PAHs), dioxins, and particulate matter, are well characterized.^4,12–16^ However, the solid by-products of OWB and other thermal oxidation processes are often overlooked.

Among these plastics, polystyrene (PS) is prominently used in single use food packaging^17–20^ and building insulation^21,22^ and is also a significant component of plastics municipal solid waste.^23,24^ The air pollutants generated during the thermal oxidation of PS and other plastics, including the particulate matter, aerosol, and volatile emissions, have been investigated thoroughly^25–27^ while the solid byproducts are understudied and are thus, the focus of this work.

Oxidative degradation of environmental PS has been of interest due to its effects on properties such as functional groups, polymer chain lengths, and bulk rigidity. Changes in these properties are relevant due to their influence on the leaching of plastic additives^28–30^ and monomers,^17,31,32^ and sorption dynamics of persistent organic pollutants.^33,34^ However, investigation of environmental oxidation has been limited to photoaging and low-temperature thermal weathering (<60 °C) induced by ultraviolet (UV) radiation, which has been extensively studied.^33,35–38^ These processes have been shown to cause polymer fragmentation into micro- and nanoplastics^35,36,39,40^ and increase the toxicity of parent PS MPs.^41–44^

Previous work on UV weathered PS identified evidence of photo-oxidative reaction mechanisms through spectroscopic techniques. These studies identified that UV radiation increased the carbonyl index of irradiated PS, or the ratio of the carbonyl (–C=O) signal to the methylene (CH_2_) scissoring signal, as measured by Fourier transform infrared (FTIR) spectroscopy, showing an increase in C–O bonds after degradation.^33,35^ Increases in the carbonyl index are indicative of the photo-oxidative degradation mechanism, wherein light initiates the production of oxygen radicals, which cause radical chain scission on the secondary carbons of the PS polymer.^36^ The termination of the reaction typically results in a terminal C=O bond, usually as a carbonyl or carboxyl group. However, in low oxygen environments, such as below the exposed surface of a particle, light energy can initiate the production of carbon radicals, which will terminate on other parts of the polymer, creating a crosslink.^45,46^ A similar phenomenon was observed for thermally oxidized PS where sites closer to the surface contained more C–O bonds, as measured by X-ray photoelectron spectroscopy.^47^ The oxygen dependency of both oxidative reaction mechanisms warrants the distinct identification of both surface and bulk chemical properties of degraded PS.

The fundamental reaction mechanism of thermal oxidation is similar to that of photo-oxidative degradation wherein, radical formation is initiated *via* heat energy rather than light.^48,49^ Higher temperatures that range from 100–700 °C, representative of heterogeneous and uncontrolled OWB and wildfires that smolder long after the initial burning event,^50^ suggest faster reaction kinetics and thus, solid by-products that will chemically differ from photo-oxidized PS. Previous work in our group showed that environmental MPs, including PS, found in the sediments of OWB sites in the Crow Reservation (MT, US) and Tuttle (OK, US) carried unique chemical signatures that differ from those of photo-oxidized plastics.^10^ The difference in chemical signature and therefore, the chemical composition, has implications for MP monitoring and its interactions with the environment. Thus, a more thorough investigation of the spectroscopic signatures of thermally oxidized PS is warranted.

The objective of this work is to use spectroscopy to identify the physical and chemical changes of PS in commercial food packaging after burning. Herein, we simulate OWB under controlled laboratory conditions for four consumer polystyrene products used for food packaging. We analyze the binding environment of carbon in the near-surface and bulk of these materials before and after burning using X-ray photoelectron spectroscopy (XPS) and attenuated total reflectance (ATR)-FTIR, respectively. The novelty of this study is the identification of changes of the functional and surface chemistry of PS caused by thermal-oxidative degradation at temperatures 350 and 425 °C. These findings will inform future risk assessments of public and environmental health regarding plastic degradation as a result of environmental thermal oxidative degradation.

## Materials and methods

### Materials

Four consumer polystyrene products were used in this study: two foam to-go boxes from two different brands (To-Go Box 1 and To-Go Box 2), a foam meat tray (Foam Tray), and a rigid bakery to-go box (Rigid Box) (WebstaruantStore, Lititz, PA, USA). The four separate products were chosen to represent a range of products that may contain different proprietary formulations.

### Preparation of burned plastics

Unburned plastics were ground with a coffee grinder^51–53^ (Krups GX322) and sieved to collect particles less than 120 µm in size. Mass loss *versus* temperature was measured for plastic powders using a thermogravimetric analyzer (TGA 5500, Waters) up to 800 °C with a ramp rate of 5 °C min^−1^ in both air and nitrogen gas (N_2_). Burning temperatures of 350 and 425 °C were chosen based on the profile of the Thermogravimetric Analysis (TGA) curves. Before burning, plastic products were cut into 1–2 cm squares using scissors that were cleaned with 70% ethanol before use. Approximately 30 mg of plastic (around 2–5 squares) was added to a clean, muffled 4 mL glass vial. Vials were initially placed in the furnace (Thermo Scientific Thermodyne) uncovered at 25 °C and set to either 350 °C or 425 °C. The vials were burned for only 20 min to prevent significant mass loss. Vials with plastics were weighed using a Mettler Toledo ME Balance before and after burning. The mass lost from each sample was less than 15% for both 350 and 425 °C (Table S2).

### Solids characterization analysis

Scanning electron microscopy (SEM) was conducted using a Tescan Vega3 XMU variable pressure SEM (Tescan Orsay Holding a.s., Brno, Czech Republic). Burned PS food packaging samples were prepared for contact angle measurements by exposing plastics to either 350 °C or 425 °C for 20 min in a glass Petri dish. After 20 min, samples were removed from the furnace and a flat glass dish was placed on top of the hot sample to create a flat surface for measurements, eliminating morphology effects that can impact contact angles.^54,55^ Samples were then cooled in a desiccator for at least one hour. Samples were fixed to a microscope slide and mounted onto a Rame-Hart 100-25-A goniometer, where contact angles were measured according to the sessile drop method with 5-µL droplets of deionized water using Rame-Hart DROPimage software.

The functional chemistry of PS samples was determined using ATR-FTIR spectroscopy (Thermo Nicolet iN10 MX) with a detection limit of 20 µm. ATR-FTIR measurements were collected with a cooled detector and germanium tip, 51 s collection time with 256 scans, spectral range of 4000–675 cm^−1^ and a resolution of 8 cm^−1^ for all samples. Aperture size was adapted to fit each examined particle. Unburned, ground powered PS or whole portions of burned PS samples were placed on Al_2_O_3_ filter for ATR-FTIR analysis; three sample points were analyzed for consistency from each sample, and one representative spectrum was selected for result reporting. Before measurements of the plastic materials, a background measurement was taken to reduce environmental noise.

XPS measurements were performed on a Kratos Ultra DLD spectrometer using a monochromatic Al Kα source operating at 150 W (1486.6 eV). The operating pressure was 5 × 10^−9^ torr. Charge compensation was accomplished using low-energy electrons. All spectra were charge referenced by adjusting the C 1s region to 285 eV. Survey spectra and high-resolution C 1s spectra were acquired at pass energies of 160 and 20 eV, respectively. XPS data were processed and deconvoluted using methods previously used in our group with Casa XPS software.^56–59^

X-ray fluorescence (XRF) spectrometry was conducted to determine bulk elemental composition using an EDAX Orbis with a Rh tube source and a titanium adsorption edge filter. Samples of plastic particles of less than 120 µm in size were measured in a vacuum with a filament voltage of 20 kV and a current of 15 µA.

## Results and discussion

### Polystyrene mass loss occurs at lower temperatures in air than in molecular nitrogen

**Fig. 1:**
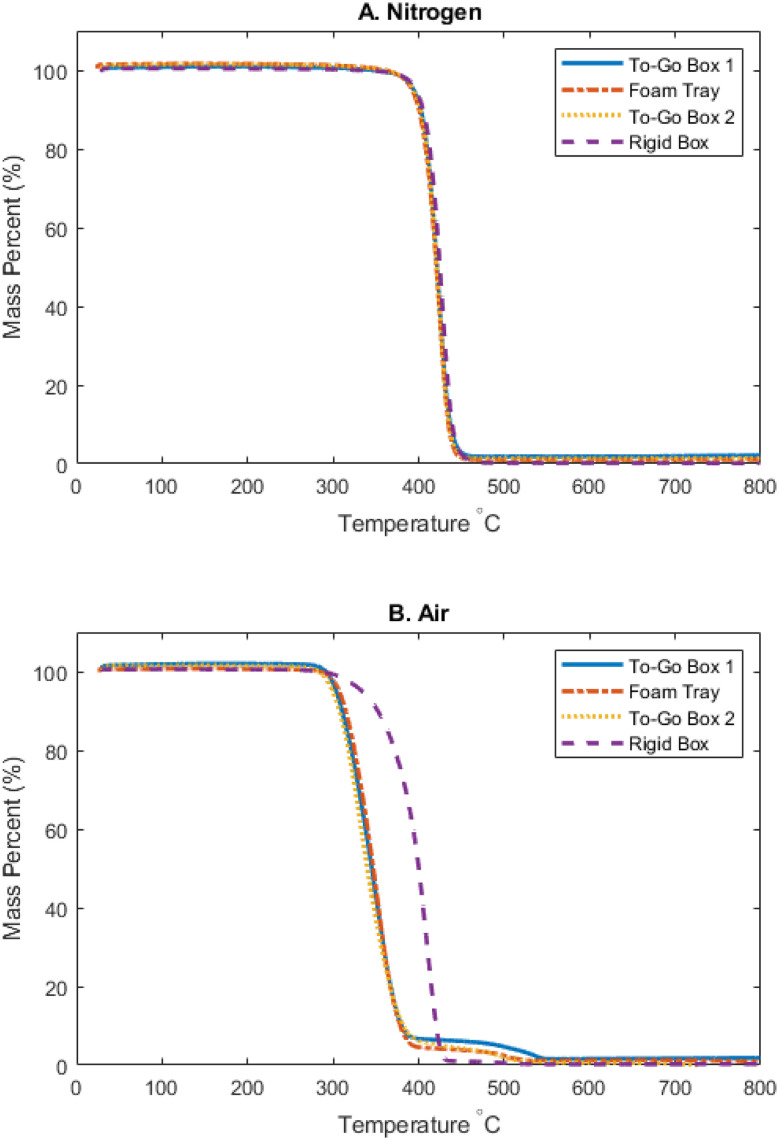
TGA of polystyrene food packaging products under nitrogen (A) or air (B) stream from 35 to 800 °C. A ramp rate of 5 °C min^−1^ was used.

The weight of all PS samples decreased rapidly starting at 400 °C in N_2_ and 350 °C in air as determined by thermogravimetric analysis (TGA) (Fig. 1). All PS samples show a nearly identical mass–loss curve in N_2_. In air, however, the mass loss curve of the Rigid Box deviates from the three foam products. In air, the three foam products exhibit a shoulder between 400 and 500 °C. The presence of this shoulder is consistent with TGA results in other studies and is often attributed to the existence of unspecified additives.^49^ As explained in the Materials and Methods subsection, burning temperatures of 350 and 425 °C were chosen based on the regions of rapid mass loss and shoulders, respectively, in the profile of the TGA curves in air.

For the foam products, the more rapid mass loss as a function of temperature in air compared to in N_2_ (Fig. 1) suggests that the O_2_ in air is accelerating the degradation of PS. Nitrogen is assumed to be inert, so in N_2_, the mass loss of PS is entirely thermally driven, with random chain scission being the primary reaction.^48^ In air, however, mass loss is caused by thermal-oxidative degradation, wherein high temperatures initiate the formation of oxygen-containing radicals that contribute to depolymerization.^49^ For the Rigid Box, no difference in mass loss as a function of temperature is observed in air compared to in N_2_, which shows that the Rigid Box may be more resistant against oxygen-containing radical species when compared to the foam products. The two burning temperatures chosen in this study are relevant due to the rapid mass loss that occurs in O_2_ around 350 °C and the shoulder exhibited around 425 °C for the three foam products.

### Polystyrene shows internal fracturing after exposure to high temperatures

**Fig. 2:**
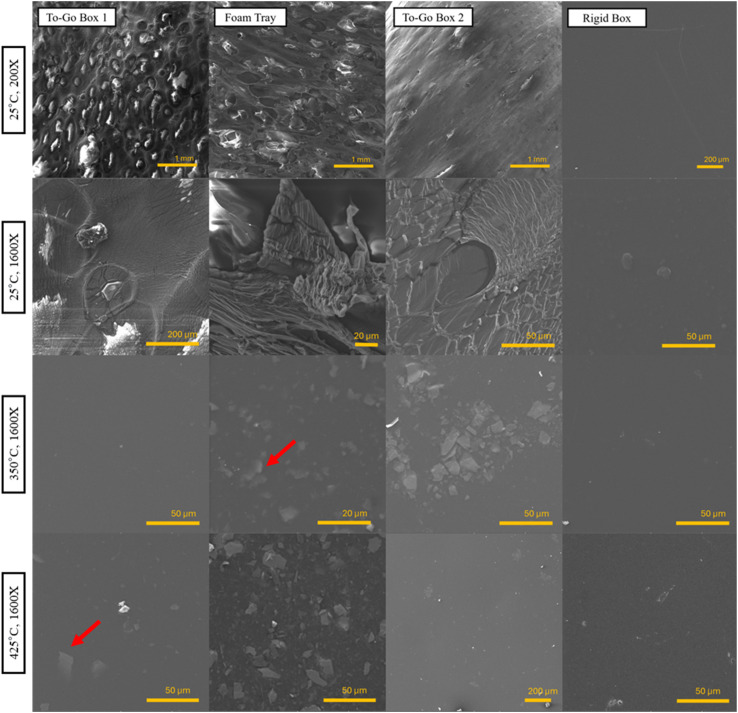
Row 1: SEM images of polystyrene products, including To-Go Box 1, Foam Tray, and To-Go Box 2 magnified at 60×, and Rigid Box magnified at 200×. Rows 2–4: SEM images at 1600× magnification of all 4 PS products: unburned (25 °C), burned at 350 °C or 425 °C for 20 min. Red arrows highlight examples of subsurface fractures.

Low-magnification SEM images of the surfaces of all four PS products before exposure to high temperatures are shown in Fig. 2. Foamed PS is manufactured by injecting air and blowing agents into PS to trap air bubbles in webs of PS films, hence the appearance of large void spaces in the three foam products (Fig. 2). The Rigid Box is noticeably different from the foam products and is devoid of notable morphological features (Fig. 2). For the ground PS MPs (Fig. S4), the ground Rigid Box particles appear to be shorter and thicker fragments, whereas the ground Foam Tray particles resemble smaller, film-like pieces.

**Fig. 3:**
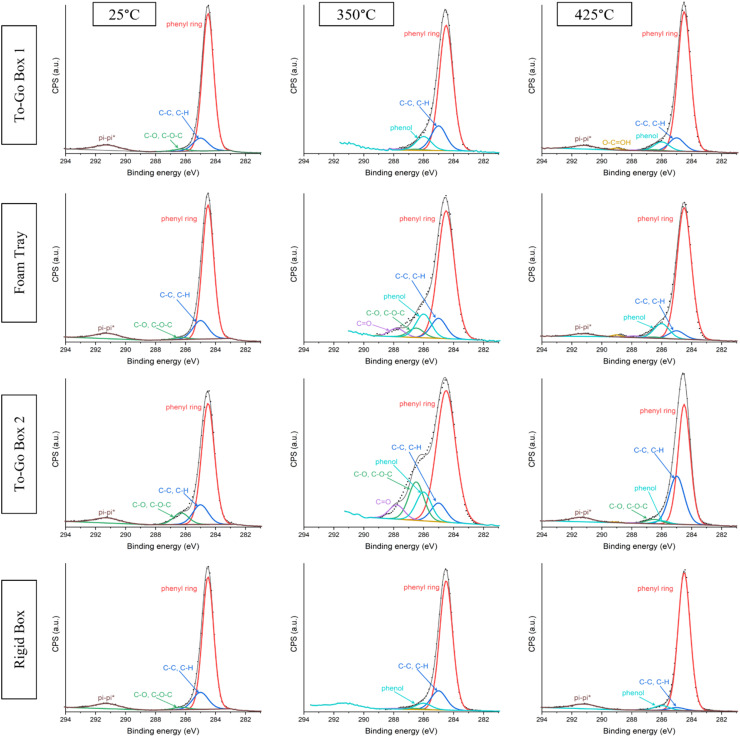
High resolution XPS C 1s spectra for polystyrene food packaging products when unburned, burned at 350 °C for 20 min, and burned at 425 °C for 20 min.

High-magnification SEM images of the surface of all four PS products are shown in Fig. 2 before after exposure to 350 and 425 °C. The surfaces of the burned foams are smooth and glossy, with few particles; however, fragmented shapes can be seen on these samples (Fig. 2), showing there is a morphological feature indicative of a fracture beneath the polymer surface. These results indicate internal cracking of the foam products after burning. This fracturing may be the result of embrittlement due to the combination of high temperatures and low temperatures for cooling.^60^ All burned PS products showed fractures except for the Rigid Box (Fig. 3).

Water contact angles are often used to assess the change in plastics' surface polarity and surface roughness after weathering. No apparent change in water contact angle was observed for any of the four plastic products after burning at either 350 or 425 °C (Fig. S3). This result differs from studies investigating other forms of oxidative weathering of PS, namely UV radiation. It has been reported that UV-aging of PS decreases water contact angles up to 39% compared to pristine PS.^61,62^ Notably, these prior studies investigated longer-term (*i.e.*, 40 days to 8 months) exposure to UV irradiation in weathering chambers, whereas in this study, we assess the impact of short-term exposure to high temperatures. These results imply that short-term exposure to high temperatures (burning) may result in less surface hydrophilization compared to longer-term exposure to UV irradiation. Importantly, surface roughness also significantly affects observed contact angles; specifically, for hydrophobic surface, increased surface roughness results in higher apparent contact angles whereas for hydrophilic surfaces, increased surface roughness results in lower apparent contact angles.^54,63^ In the current study, after burning, all samples appear relatively smooth (Fig. 2). Prior studies that observed lower contact angles after long-term UV aging also observed increased surface roughness of PS particles through surface micro-cracking and fragmentation.^36,61,62^ For a hydrophilized PS particle surface, this increased surface roughness could also result in lower apparent contact angles, separate from changes in surface chemistry (polarity). Because the measured water contact angles do not distinguish between changes in surface morphology *versus* surface chemistry (polarity), spectroscopic techniques were used to further assess surface polarity of burned PS samples in this study.

The atomic percentages of P, Ca, Fe, and Zn detected by XRF show a similar elemental composition in all four PS materials used in this study (Fig. S2). Ca and P were the most abundant elements in all PS products detected by XRF. These elements can be added to plastics as metallic and organophosphate plastic additives which serve as heat stabilizers, antioxidants, fillers, and pigments.^64–66^ However, the lack of enrichment of a particular element in any of the four plastics as indicated by XRF analyses suggests that a specific metallic or organophosphate heat stabilizing additive is not the reason that the Rigid Box is particularly resistant to thermal-oxidative degradation.

### Spectroscopic identification by XPS of surface C–O bonds on PS after burning

The detection of shoulders in the range of 285 to 289 eV in all PS product spectra aby XPS analyses after burning at either 350 °C or 425 °C for 20 min indicates the presence of C–O bonds to the surface as a result of thermal oxidative degradation (Fig. 3). The primary C–C peak was split into two separate peaks for phenyl and aliphatic groups based on binding energy values in Beamson and Briggs, 1993.^67^ Similar shoulders greater than 285 eV have been reported after exposure to 120 °C for one hour.^47^ For unburned (25 °C) samples, any peak associated with a C–O bond likely corresponds to an impurity or additive in the plastic. For instance, To-Go Box 2 contains the greatest area associated with C–O bonds for all the unburned samples, which indicates that it may contain more impurities or additives than other plastic products (Table 1). For burned samples, fits of spectra to different C–O bond types suggest that burned PS samples contained a higher atomic percentage and greater variety of C–O bonds compared to the unburned samples. The greatest peak for all spectra is the phenyl peak, which is attributed to the aromatic carbons in the PS molecular structure.

**Table 1:** Percent distributions corresponding to fitting of high-resolution XPS C 1s spectra for PS food packaging products when unburned, burned at 350 °C for 20 min, and burned at 425 °C for 20 min

Plastic	Temp (°C)	C phenyl ring (%)	C–C, C–H (%)	C phenol (%)	C–O, C–O–C (%)	C=O (%)	O–C=OH (%)
To-Go Box 1	25	76.2 ± 10.7	21.2 ± 9.8	0.0 ± 0.0	2.6 ± 0.9	0.0 ± 0.0	0.0 ± 0.0
	350	75.3 ± 3.0	13.1 ± 3.5	8.5 ± 2.2	1.8 ± 2.0	1.3 ± 1.0	0.0 ± 0.0
	425	77.6 ± 6.2	11.0 ± 2.2	9.0 ± 3.6	0.8 ± 0.1	0.7 ± 0.4	0.9 ± 0.1
Foam Tray	25	87.8 ± 6.7	10.0 ± 7.4	0.0 ± 0.0	2.2 ± 0.8	0.0 ± 0.0	0.0 ± 0.0
	350	72.9 ± 6.8	9.6 ± 2.0	11.5 ± 3.6	3.9 ± 3.5	2.1 ± 2.0	0.0 ± 0.1
	425	78.1 ± 3.5	7.5 ± 1.7	12.2 ± 2.1	0.7 ± 0.4	0.9 ± 0.2	0.7 ± 0.2
To-Go Box 2	25	80.2 ± 5.4	11.9 ± 4.2	0.0 ± 0.0	7.9 ± 1.2	0.0 ± 0.0	0.0 ± 0.0
	350	66.4 ± 6.1	6.8 ± 0.4	14.0 ± 4.5	9.3 ± 7.4	3.4 ± 2.7	0.0 ± 0.0
	425	74.2 ± 15.9	18.9 ± 18.0	4.6 ± 3.8	1.4 ± 2.0	0.4 ± 0.1	0.5 ± 0.3
Rigid Box	25	82.1 ± 1.1	15.6 ± 0.9	0.0 ± 0.0	2.3 ± 0.3	0.0 ± 0.0	0.0 ± 0.0
	350	78.6 ± 1.5	12.7 ± 1.7	7.2 ± 2.2	1.0 ± 0.8	0.6 ± 0.1	0.0 ± 0.0
	425	84.8 ± 11.9	8.3 ± 8.2	5.3 ± 1.7	0.8 ± 1.1	0.2 ± 0.1	0.7 ± 0.8

Generally, the relative size of shoulders greater than 285 eV in the C 1s spectra is greater after burning at 350 °C for 20 min, indicating that oxidation is greatest for all plastics after burning at 350 °C for 20 min (Fig. 3). Comparing across all plastics, the highest intensity for the shoulder located in the range of 285 to 289 eV was detected in To-Go Box 2 after burning at 350 °C. Fitting suggests that the oxygenated bonds on the surface include C–O, phenolic, and C=O bonds. The highest intensity of shoulders corresponding to oxidized carbon bonds was also identified in the To-Go Box 2 sample burned at 350 °C. For To-Go Box 2, the intensity of the shoulder located in the range of 285 to 289 eV was lowest after burning at 425 °C for 20 min. Based on fitting of high-resolution C 1s XPS spectra, we identified an increase in aliphatic bonds and decrease in the phenylic bonds at 425 °C when compared to the unburned and burned (350 °C) To-Go Box 2 samples.

Interestingly, for the Rigid Box, high resolution XPS analyses reveal limited changes in the spectra as a function of burning temperature when compared to the three foam samples. The total atomic percentage associated with C/O bonds in the Rigid Box remains the lowest of the four products among each respective temperature. The lack of C–O bond signals in the Rigid Box after burning at both 350 and 425 °C suggests that the Rigid Box is more resistant to oxidative degradation than the other plastics. This observation is also supported by the results for weight loss TGA curves in air (Fig. 1). As discussed previously, this thermal stability is likely not the result of heat-resistant additives, since the characteristic shoulder (400–500 °C) that is associated with additives exhibited by the three foam plastics is missing from the Rigid Box TGA curve (Fig. 1). Future work should determine the impacts of physical properties on PS heat stability.

Fittings of high-resolution C 1s XPS spectra indicate that phenyl ring contains the greatest area across all temperatures and all four plastic products. It is possible that other aromatic compounds are forming at the surface but are indistinguishable from phenyl based on the binding energies of carbon in those products. It is unlikely that carbonization of PS will occur at these temperatures, but burning of PS can produce many difference aromatic carbon species such as carbon black and PAHs.^6,15,25^ The binding energies of a carbon in black carbon are expected to be similar to a carbon in a phenyl group of styrene, and therefore, indistinguishable from one another.^68^

### Changes in functional chemistry identified by FTIR after polymer degradation from burning

**Fig. 4:**
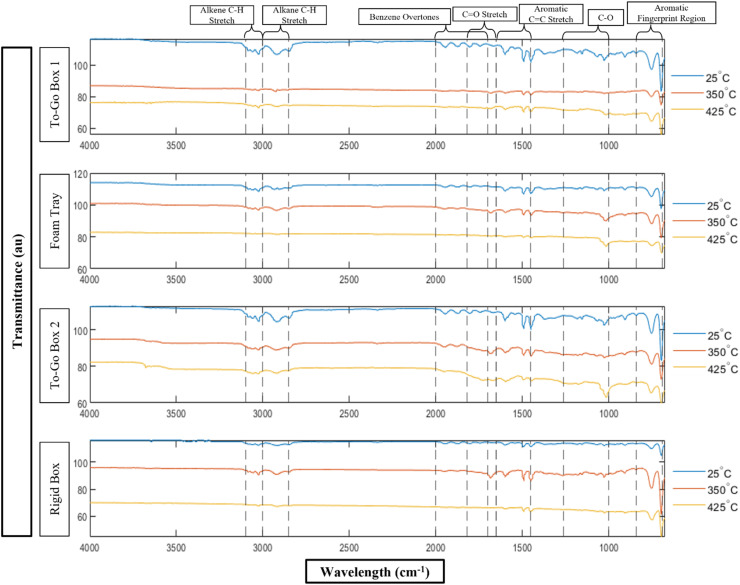
FTIR spectra for polystyrene food packaging products when unburned (25 °C), burned at 350 °C for 20 min, and burned at 425 °C for 20 min. Note that wavelength intervals of interest are shown with dashed lines.

FTIR analyses revealed changes in the IR spectra in all samples of the burned PS food packaging products when compared to the respective reference spectra (unburned or 25 °C) (Fig. 4). The major wavelength regions associated with the PS polymer are the alkane C–H stretch region (3000–2840 cm^−1^), the aromatic C=C region (1675–1475 cm^−1^), and the aromatic sp^2^ C–H region (1000–840 cm^−1^).^69^ For the three foam products (To-Go Box 1, To-Go Box 2, and Foam Tray), the peaks in all three of these regions decrease as temperature increases. These changes in spectra suggest degradation of the PS polymer as a result of burning.^70^ For the Rigid Box, all the peaks in these regions increase at 350 °C and then decrease at 425 °C.

Evidence of oxidation can be observed in the C=O stretch (1818–1650 cm^−1^)^71^ and the C–O (1260–1000 cm^−1^) regions for all plastic products (Fig. 4). However, there is a more notable change in spectra for the C–O regions than the C=O region, showing less carbonyl formation. This result is different from the results found for UV weathering of PS, where peaks in the carbonyl (C=O) region significantly increase after weathering.^35,62^ For the Foam Tray and To-Go Box 2, the peaks in the C–O region increase with increasing burning temperature, where peaks have the highest intensity at 425 °C. This result contrasts with the results obtained from XPS, where the greatest intensities associated with C/O bonds were observed at 350 °C. The difference in trends between the IR and XPS spectra as temperature increases suggests that the extent of oxidation can differ between the bulk and the surface of the plastic.

The peaks in both C=O (1818–1650 cm^−1^) and C–O (1260–1000 cm^−1^) bond regions for the Rigid Box at all temperatures are smaller than the peaks in the same regions of each foam products' spectrum. The intensities of the peaks for the Rigid Box's spectrum are greatest for the sample burned at 350 °C. This trend suggests that the Rigid Box starts showing signs of degradation at higher temperatures compared to the foam samples.

### Insights from thermal oxidative degradation of food packaging PS

Thermal degradation can be deduced from changes in spectra observed at the bulk and surface level. Many oxidative PS degradation models include reactions that occur mostly at the aliphatic chain rather than the aromatic ring. During thermal degradation, the initiation of O radicals will produce hydroperoxides on the 3° carbons. Further reaction of these hydroperoxides will lead to chain scission and the formation of a carbonyl group (C=O).^72^ This pathway conflicts with our results because the signals for both C=O in XPS spectrum and C=O in FTIR spectrum are weak. Instead, most of the C/O signals detected using both XPS and FTIR are associated with C–O, rather than C=O. Fitting of shoulders greater than 285 eV detected in high resolution C 1s spectra suggests that most C/O bonds on the surface of all four plastics after burning are phenolic or other C–O bonds. The formation of phenolic compounds from exposure to temperatures between 100 and 600 °C is possible, but unlikely, due to the high energy input needed to break the bonds in the aromatic ring.^72^ The temperatures (350 °C or 425 °C) used in our study may be sufficient to provide the necessary energy input, leading to the formation of phenols on the surface of burned PS.

Despite being present on the surface, there is not much indication of phenolic compounds being present in the bulk material. The distinct alcohol group signature (peak at 3600 cm^−1^), which is typically present for UV weathered PS,^62^ is absent from IR spectra of all burned and unburned plastics. This suggests that degradation products will be dependent on O content, leading to differences in the bulk and surface of burned PS plastics. This result is consistent with other weathering studies including PS and other polymers, where oxidation is inversely correlated to surface depth.^47,62^ Similarly, the dominant photo-oxidative degradation mechanism in polyolefins is also dependent on the O content in the polymer matrix.^46^ At the surface, in the presence of oxygen, polyolefin degradation was dominated by chain scission, where the bulk material experienced more crosslinking.

Crosslinking of PS products as a result of burning is suggested by the decrease in aliphatic bond fit intensities from C 1s spectra and increase in C/O single bond peaks in IR spectra. Crosslinking of either the 2° or 3° carbons would change the binding energy of that aliphatic carbon to be similar to that of a phenylic carbon, hence the general conservation of the phenyl peak fit in the C 1s spectra. It is also possible that this crosslinking occurs at the benzene ring. The change in the benzene overtones or “benzene fingers” detected from 2000–1700 cm^−1^ suggests that the mono-substituted aromatic rings of PS are becoming poly-substituted with additional bonds. The distinct fingerprint of a monosubstituted benzene is shown as four short, equidistant peaks in this region^69,73^ in the FTIR spectra for all four unburned plastics (Fig. 4). These overtones flatten, and shift in some cases, for all PS products as the burning temperature increases. The decrease or shift of these overtones (as in the case of To-Go Box 2) indicates that the benzene rings are becoming di-substituted. This result, paired with the increasing C–O IR signal of the burned plastics, suggests that the C–O single bonds may be serving as the crosslink between aromatic rings. Evidence of crosslinking is also observed in SEM imaging of burned plastics showing subsurface fractures. Crosslinking can cause PS and other plastics to become brittle;^71,74^ therefore, the internal fractures observed in SEM images (Fig. 2) may be a result of embrittlement from crosslinking. In future work, gel permeation chromatography should be performed to quantify the extent of crosslinking in unburned and burned PS.

## Conclusions

Herein, we identified the spectroscopic changes of four PS food packaging products after burning at 350 and 425 °C to better understand the degradation mechanisms of PS at temperatures that are representative of environmental thermal oxidation. These temperatures are particularly relevant for OWB and the burning of residential buildings at the urban–wildfire interface. XPS C 1s spectra showed that the surface of PS became oxidized with the addition of C/O bonds after burning. However, FTIR spectra of the bulk material exhibited a lesser degree of oxidation and evidence of polymer chain scission and crosslinking, demonstrating the oxygen dependence of the thermal oxidation mechanism. These spectroscopic changes will impact interactions between burned PS and the environment, making these results relevant for informing future environmental health risks. Additionally, FTIR spectra of burned PS were missing key features found in spectra of UV weathered PS, which could have implications for monitoring of environmental MPs. Further investigations should be conducted to quantify the impact of burning on the leaching of organic additives and oligomers from PS and other plastics.

## Author contributions

Maycee Hurd: investigation, methodology, visualization, writing – original draft, writing – review and editing. Xuewen Wang: investigation and formal analysis. Angelica Benavidez: investigation, formal analysis, and visualization. Allyson L. McGaughey: methodology, conceptualization, writing – review and editing. Michael Spilde: investigation and formal analysis. José M. Cerrato: conceptualization, methodology, supervision, resources, writing – review and editing. Jorge Gonzalez-Estrella: conceptualization, writing – original draft, writing – review and editing. Eliane El Hayek: conceptualization, supervision, resources, project administration, writing – review and editing.

## Conflicts of interest

The authors declare no conflicts of interest.

## Supplementary Material

VA-005-D5VA00291E-s001

## Data Availability

All data is available in the supplementary information (SI) file. Supplementary information: three tables and ten figures are available in the SI. See DOI: https://doi.org/10.1039/d5va00291e.
