# Supplementary material for: Spectroscopic investigation of the effects of simulated open waste burning on the functional and surface chemistry of commercial polystyrene
Source: Env Sci Adv. 2026 Mar 9;5(4):1050–9. doi: 10.1039/d5va00291e (PMC12970243; doi:10.1039/d5va00291e)
Supplement: VA-005-D5VA00291E-s001 [file VA-005-D5VA00291E-s001.pdf]

Supplementary Information (SI) for Environmental Science: Advances.
This journal is © The Royal Society of Chemistry 2026

Supporting Information:

Spectroscopic Investigation of the Effects of Simulated Open Waste Burning on the Functional and Surface Chemistry of Commercial Polystyrene

Maycee Hurd[1,5‡], Xuewen Wang[2], Angelica Benavidez[3], Allyson L. McGaughey[1], Mike Spilde[4], José M. Cerrato[1], Jorge Gonzalez-Estrella[2], and Eliane El Hayek[5]*

[1] Gerald May Department of Civil, Construction & Environmental Engineering, MSC01 1070, University of New Mexico, Albuquerque, New Mexico 87131, USA
[2] School of Civil & Environmental Engineering, 248 Engineering North, Oklahoma State University, Stillwater, Oklahoma, USA 74078
[3] Center for Micro Engineered Materials, 1001 University Dr., Albuquerque, New Mexico 87106, USA
[4] Department of Earth and Planetary Sciences, MSC03 2040, University of New Mexico, Albuquerque, NM 87131, USA
[5] Department of Pharmaceutical Sciences, MSC09 5360, University of New Mexico, College of Pharmacy, Albuquerque, New Mexico 87131, USA
[‡] Department of Civil and Environmental Engineering, Carnegie Mellon University, Pittsburgh, Pennsylvania 15213, USA

*Corresponding author: Maycee Hurd (mhurd@andrew.cmu.edu)

Summary: 13 pages, 3 tables, 10 figures.

Table of Contents

Table S1. Product Description for Plastic Types used in this study. 3

Table S2. Mass Loss of plastic samples after burning at 350°C and 425°C for 20 mins. 4

Table S3. Atomic percentages of C, Ca, O, and Si for all 4 plastics at 25°C (unburned). 6

Figure S3. Water contact angle measurements for all 4 plastics when unburned, 350°C for 20 mins, and 425°C for 20 mins. At least 8 measurements taken per sample. 7

Figure S4. SEM images of Foam Tray and Rigid box particles ground to less than 120 µm. Magnification 200X. 8

Figure S5. SEM images of Foam Tray Particles less than 120 um at 260x, 1600x, and 4000x magnification. 9

Figure S6. SEM images of rigid box particles less than 120 um at 260x, 1600x, and 4000x magnification. 9

Figure S7. SEM images of Foam Tray. SEM images of To-Go Box 2. Top row contains unburned samples, middle row contains samples burned at 350°C for 20 min, and bottom contains samples burned at 425°C for 20 min. 10

Figure S8. SEM images of the Rigid Box. SEM images of To-Go Box 2. Top row contains unburned samples, middle row contains samples burned at 350°C for 20 min, and bottom contains samples burned at 425°C for 20 min. 11

Figure S9. SEM images of To-Go Box 1. SEM images of To-Go Box 2. Top row contains unburned samples, middle row contains samples burned at 350°C for 20 min, and bottom contains samples burned at 425°C for 20 min. 12

Figure S10. SEM images of To-Go Box 2. Top row contains unburned samples, middle row contains samples burned at 350°C for 20 min, and bottom contains samples burned at 425°C for 20 min. 13

Table S1. Product Description for Plastic Types used in this study.

Plastic Product	*Brand and Product Number*	*Product Description*	*Shorthand Abbreviation*
To-Go Box 1	Dart 90HTPF1R	9" x 9" x 3" White Foam Square Take Out Container with Hinged Lid	DT
To-Go Box 2	Genpak SN203-WHT	9 1/4" x 9 1/4" x 3" White Large 3-Compartment Foam Snap-It Hinged Lid Container	GP
Foam Tray	CKF 88103 (#2S)	White Foam Meat Tray 8 1/4" x 5 3/4" x 1/2"	TR
Rigid Box	Dart C57PST1	ClearSeal Hinged Lid Plastic Container 6" x 5 13/16" x 3"	RG

Table S2. Mass Loss of plastic samples after burning at 350°C and 425°C for 20 mins.

Sample	*Vial Wt. (g)*	*Vial + PS (g)*	*Vial +PS after burn (g)*	*Mass Loss %*
**25°C (no burn)**				
**Control**	4.773	4.773	4.773	-
**To-Go Box 1**	4.78	4.814	4.814	-
**Foam Tray**	4.816	4.851	4.851	-
**To-Go Box 2**	4.78	4.814	4.814	-
**Rigid Box**	4.784	4.819	4.819	-
**350°C**				
**Control**	4.778	4.778	4.778	-
**To-Go Box 1**	4.774	4.812	4.81	7
**Foam Tray**	4.78	4.816	4.814	4.1
**To-Go Box 2**	4.787	4.81	4.81	2.6
**Rigid Box**	4.781	4.82	4.819	2.1
**425°C**				
**Control**	4.785	4.785	4.785	-
**To-Go Box 1**	4.775	4.84	4.832	12.8
**Foam Tray**	4.779	4.833	4.829	7.8
**To-Go Box 2**	4.783	4.845	4.842	6.1
**Rigid Box**	4.784	4.832	4.829	4.4

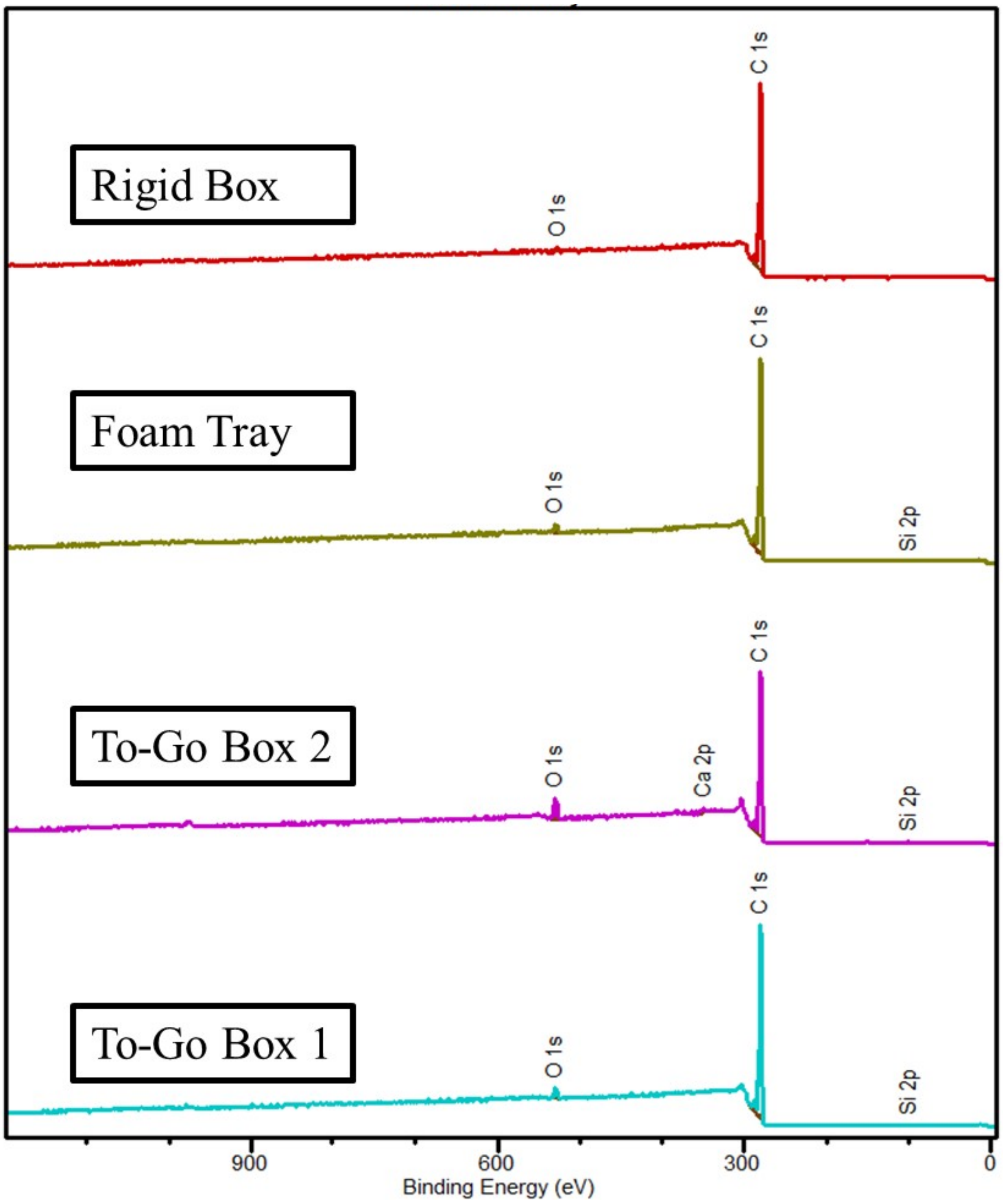

Figure S1. XPS survey scans for all the Rigid Box, Foam Tray, To-Go Box 1, and To-Go Box 2.

Table S3. Atomic percentages of C, Ca, O, and Si for all 4 plastics at 25°C (unburned).

Plastic Product	***C 1s%***	***Ca 2p%***	***O 1s%***	***Si 2p%***
Rigid Box	96.15	0.00	2.82	0.83
Foam Tray	95.15	0.00	3.17	1.68
To-Go Box 1	96.08	0.00	3.55	0.37
To-Go Box 2	94.40	0.20	4.54	0.85

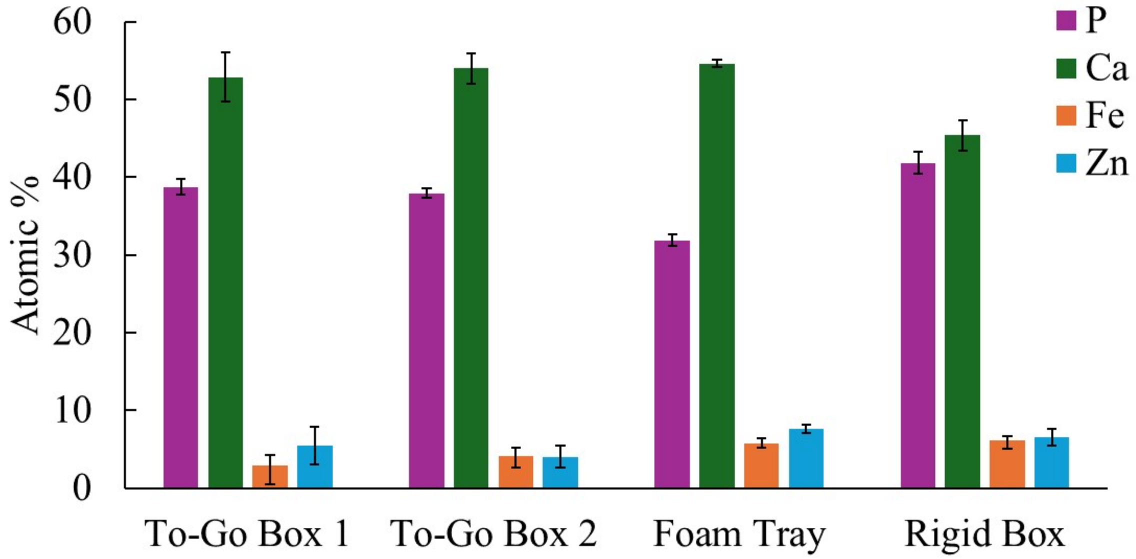

Figure S2. Atomic percentages of P, Ca, Fe, and Zn for all 4 plastics at 25°C (unburned) obtained with XRF.

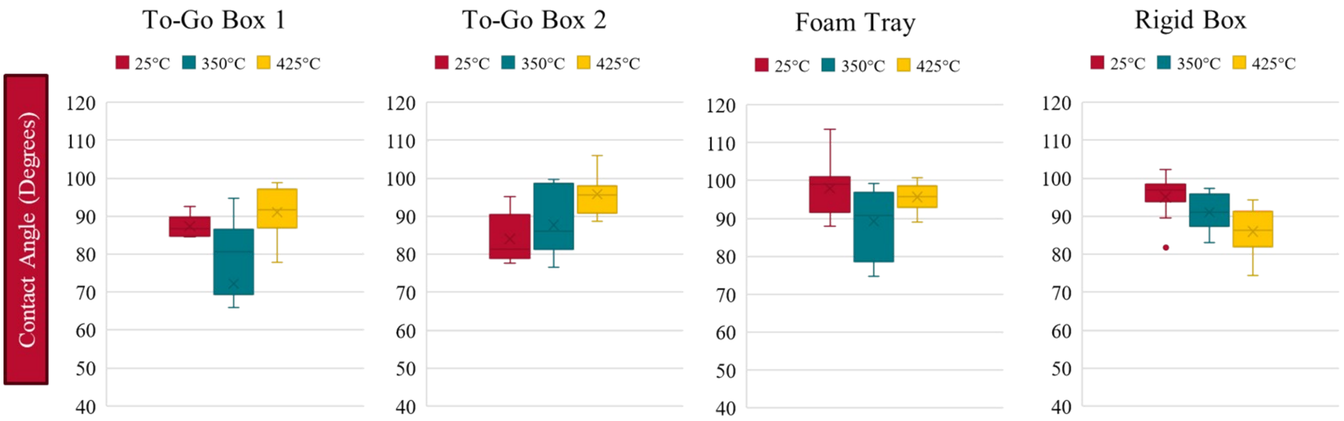

Figure S3. Water contact angle measurements for all 4 plastics when unburned, 350°C for 20 mins, and 425°C for 20 mins. At least 8 measurements taken per sample.

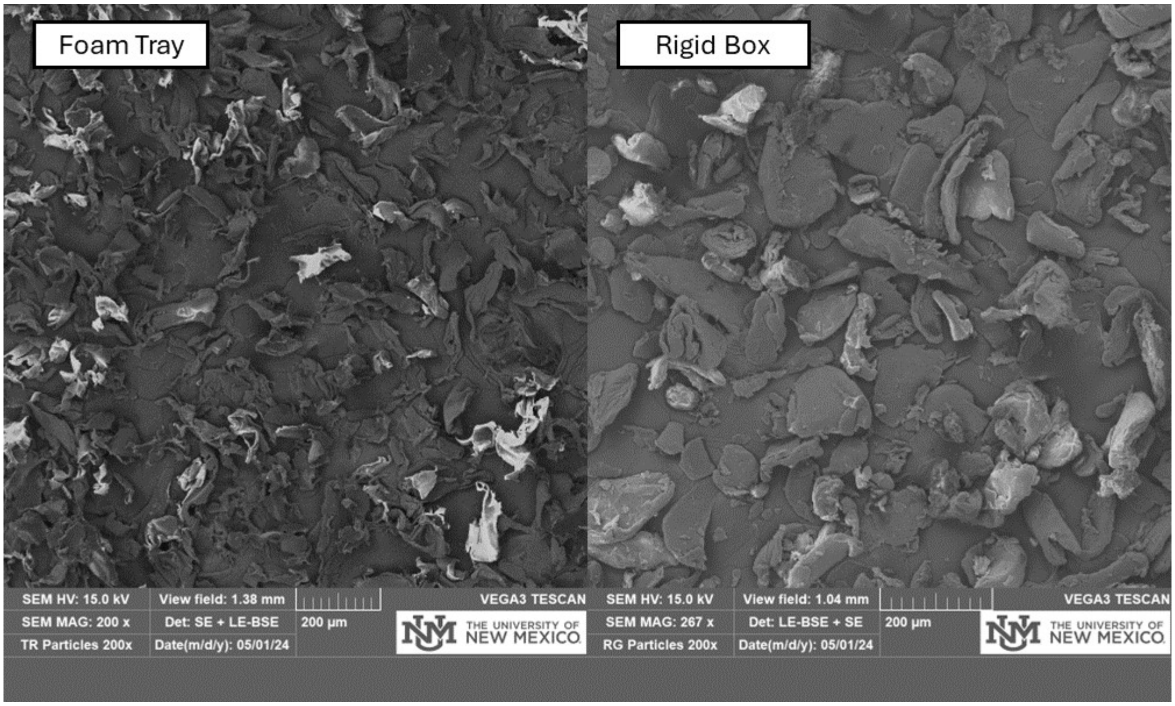

Figure S4. SEM images of Foam Tray and Rigid box particles ground to less than 120 µm. Magnification 200X.

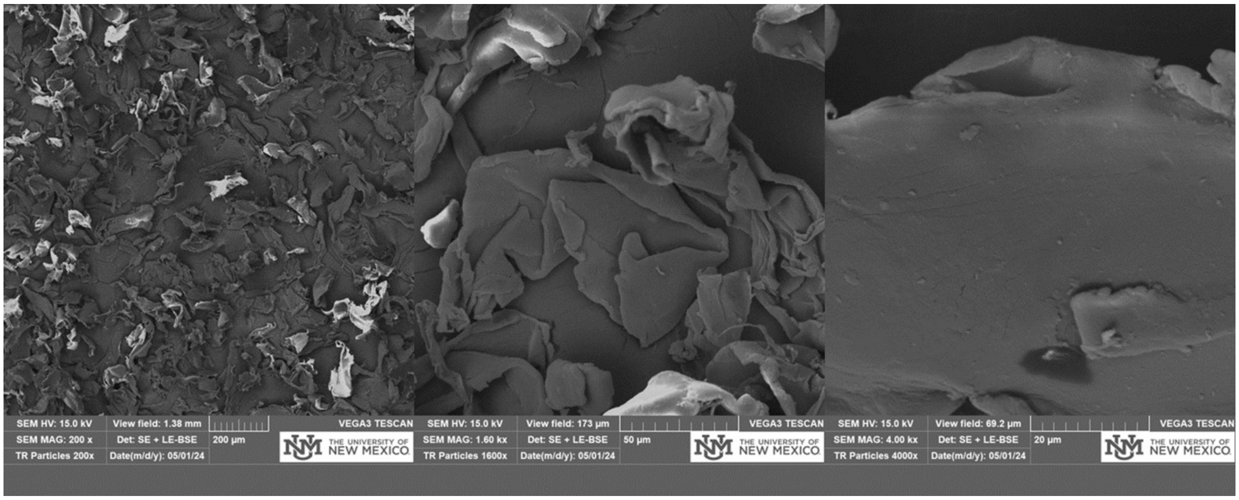

Figure S5. SEM images of Foam Tray Particles less than 120 um at 260x, 1600x, and 4000x magnification.

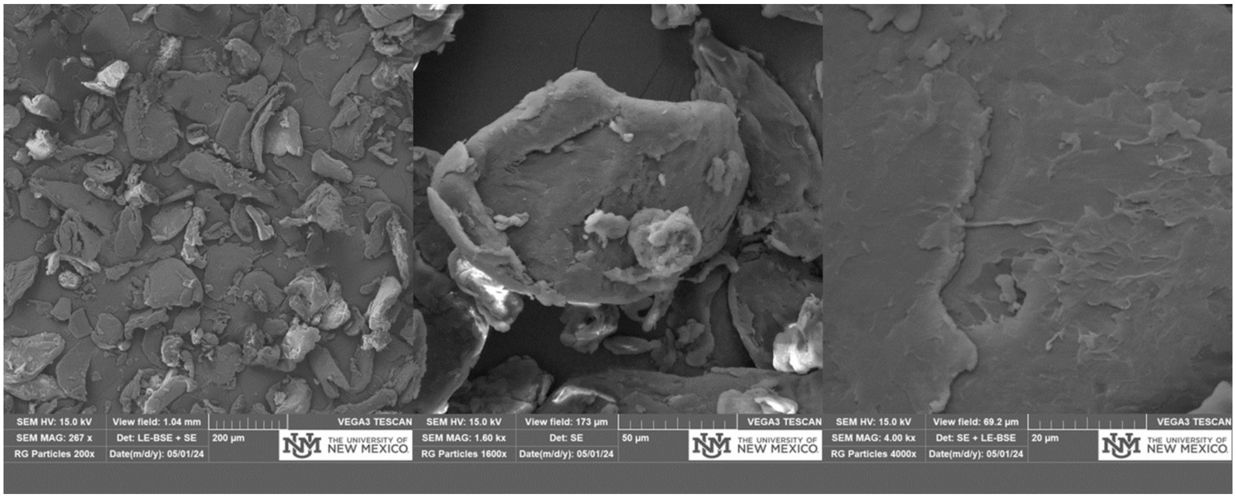

Figure S6. SEM images of rigid box particles less than 120 um at 260x, 1600x, and 4000x magnification.

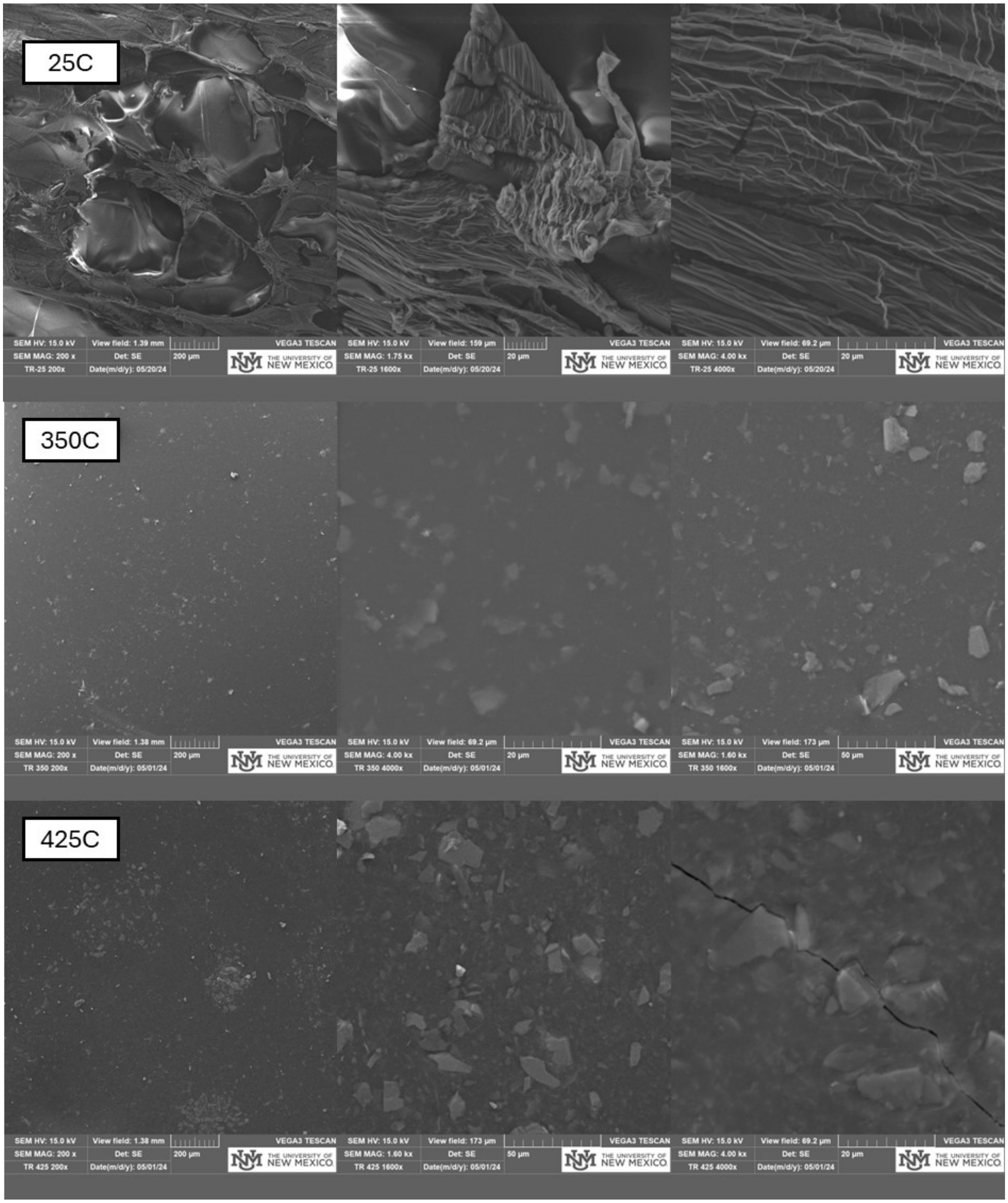

Figure S7. SEM images of Foam Tray. SEM images of To-Go Box 2. Top row contains unburned samples, middle row contains samples burned at 350°C for 20 min, and bottom contains samples burned at 425°C for 20 min.

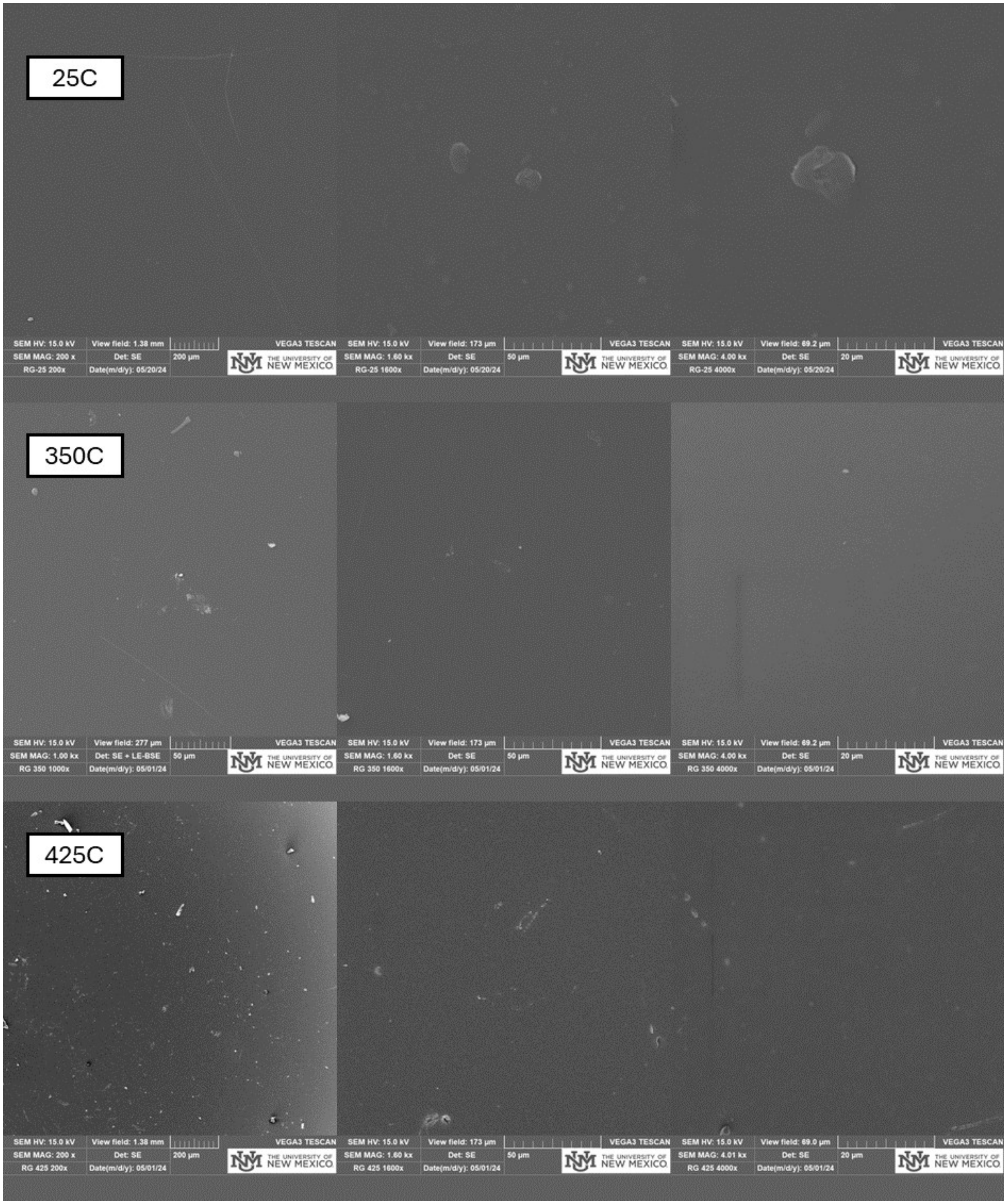

Figure S8. SEM images of the Rigid Box. SEM images of To-Go Box 2. Top row contains unburned samples, middle row contains samples burned at 350°C for 20 min, and bottom contains samples burned at 425°C for 20 min.

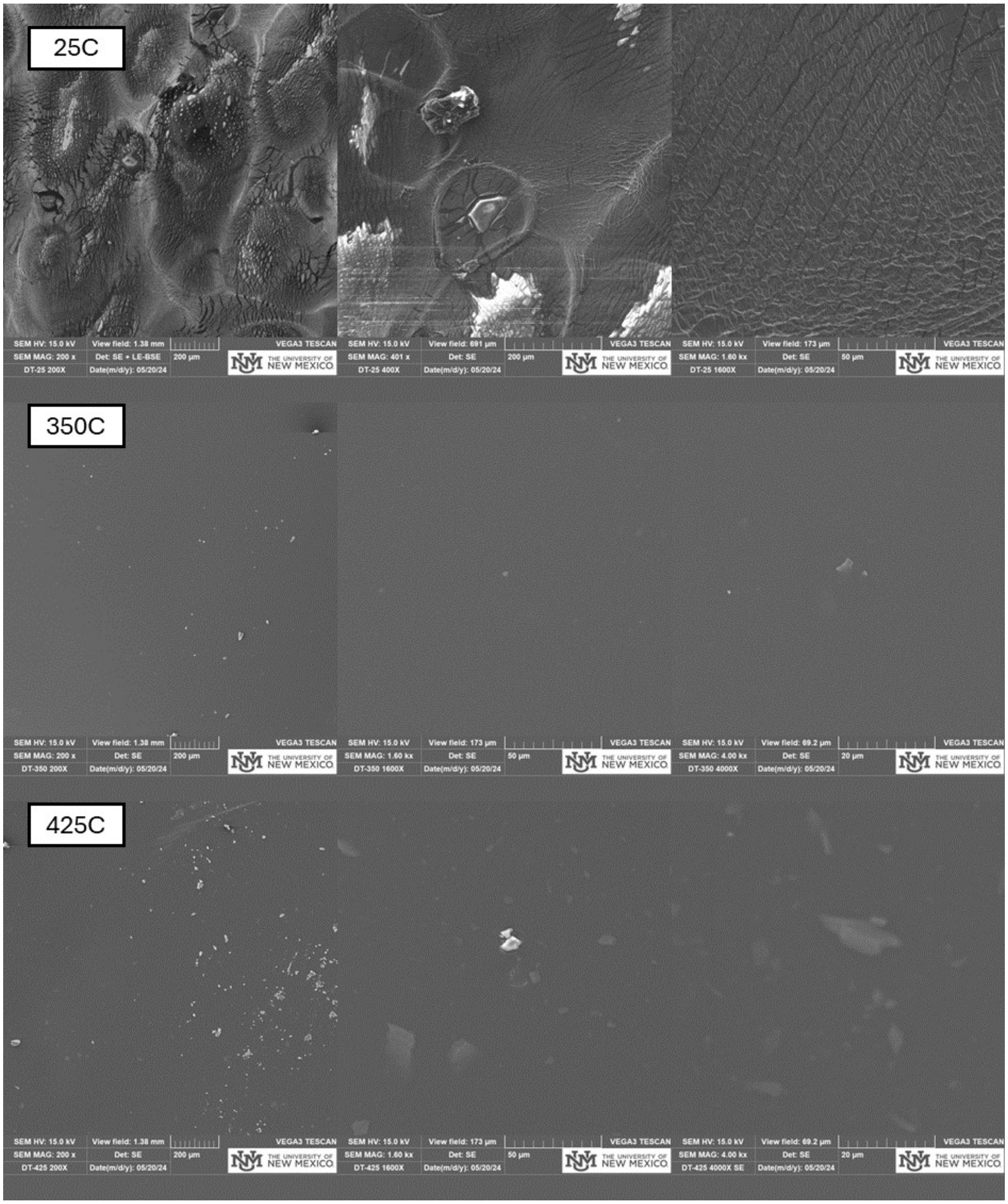

Figure S9. SEM images of To-Go Box 1. SEM images of To-Go Box 2. Top row contains unburned samples, middle row contains samples burned at 350°C for 20 min, and bottom contains samples burned at 425°C for 20 min.

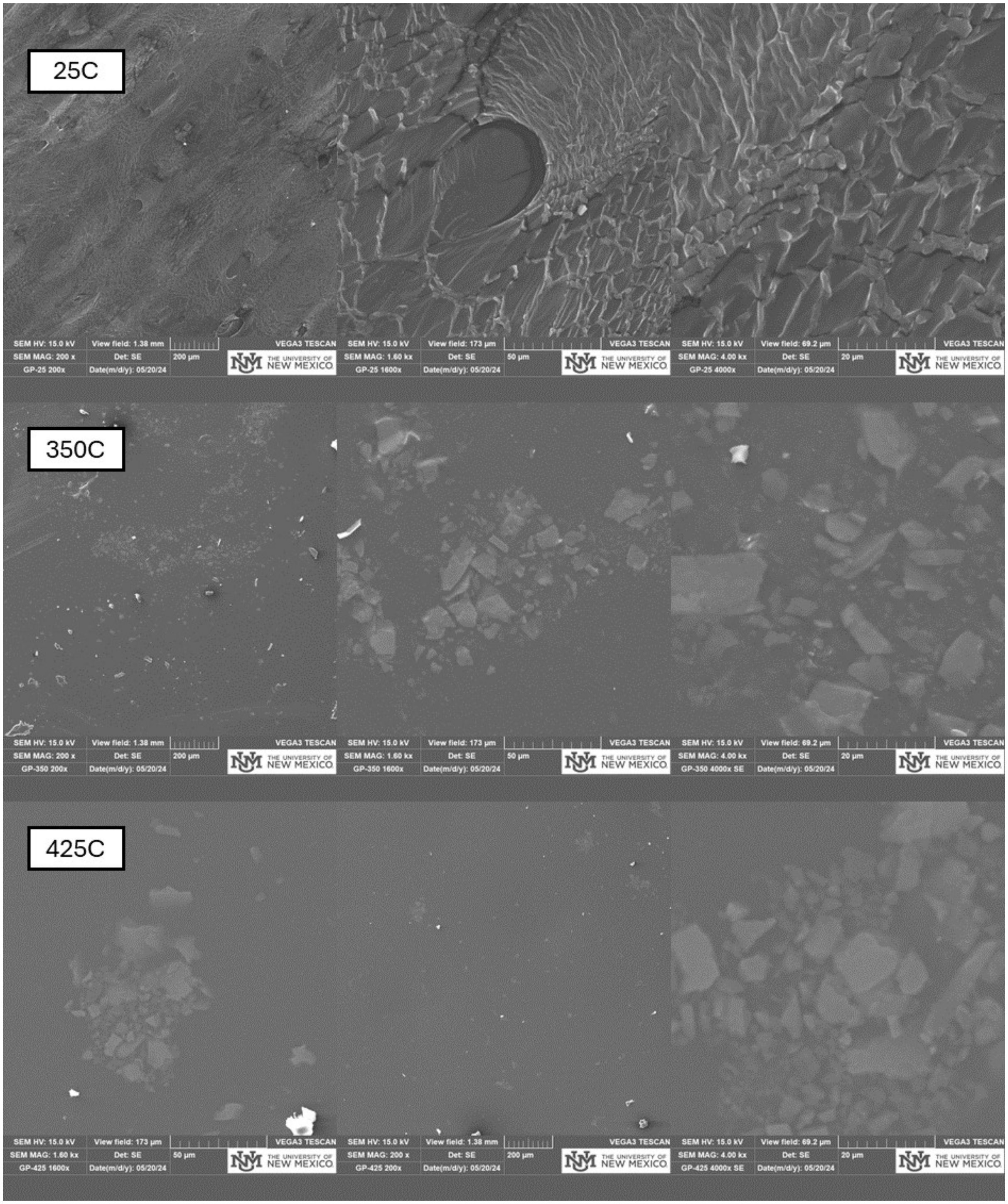

Figure S10. SEM images of To-Go Box 2. Top row contains unburned samples, middle row contains samples burned at 350°C for 20 min, and bottom contains samples burned at 425°C for 20 min.
